# Infection and Pulp Regeneration

**DOI:** 10.3390/dj4010004

**Published:** 2016-03-10

**Authors:** Sahng G. Kim

**Affiliations:** Division of Endodontics, Columbia University College of Dental Medicine, 630 W. 168th St. PH7Stem #128, New York, NY 10032, USA; sgk2114@columbia.edu; Tel.: +1-212-305-4594

**Keywords:** infection, disinfection, pulp regeneration, regenerative endodontics, the pulp-dentin complex, dentinal tubule, immune response, biofilm

## Abstract

The regeneration of the pulp-dentin complex has been a great challenge to both scientists and clinicians. Previous work has shown that the presence of prior infection may influence the characteristics of tissues formed in the root canal space after regenerative endodontic treatment. The formation of ectopic tissues such as periodontal ligament, bone, and cementum has been observed in the root canal space of immature necrotic teeth with apical periodontitis, while the regeneration of dentin and pulp has been identified in previously non-infected teeth.  The current regenerative endodontic therapy utilizes disinfection protocols, which heavily rely on chemical irrigation using conventional disinfectants. From a microbiological point of view, the current protocols may not allow a sufficiently clean root canal microenvironment, which is critical for dentin and pulp regeneration. In this article, the significance of root canal disinfection in regenerating the pulp-dentin complex, the limitations of the current regenerative endodontic disinfection protocols, and advanced disinfection techniques designed to reduce the microorganisms and biofilms in chronic infection are discussed.

## 1. Introduction

All regenerative endodontic procedures are based on complex biological and physiological processes of tissue regeneration, which are closely associated with therapeutic outcomes. To investigate the outcomes of this new treatment modality adopted by American Dental Association in 2011, there have been many clinical attempts including case reports/series [1,2,3,4,5,6] and clinical outcome studies [7,8,9,10,11] using a variety of different protocols. On the basis of the outcomes of clinical trials, the therapeutic effects of regenerative endodontic treatment have been demonstrated to be a favorable alternative to conventional root canal treatment [7,8,9,10,11]. However, histological findings have not shown robust regeneration in previously infected teeth [12,13,14].

Despite the recent explosion of publications on dental pulp-dentin regeneration, questions concerning how differently our immune system responds to the regenerative procedures than to conventional root canal treatment in a microbiological and regenerative context remain unresolved—for example, how significantly the presence of remaining microorganisms in the root canal space after disinfection procedures affects the outcomes. Furthermore, the importance of creating a microenvironment in the disinfected root canal system that is conducive to the formation of new dentin and pulp rather than tissues of periodontal origin has not been well studied. This review will discuss the dentrimental effect of prior infection on the regeneration of the pulp-dentin complex, the significance of dentin microstructure as a geometrical cue for the migration and differentiation of stem/progenitor cells, the limitations of non-selective current disinfection regimens, and newer disinfection techniques designed to eradicate microorganisms and biofilms in chronic infection.

## 2. Prior Infection and Tissue Healing

Tissue healing, whether it is repair or regeneration, occurs in a sterile or highly disinfected microenvironment where host immune defense system does not promote tissue-destructive proinflammatory processes but can stimulate tissue-forming processes to replace inflammatory tissues with native or ectopic tissues [15]. It is suggested that the determination of the tissues formed during the wound healing process strongly depends on a multitude of local parameters such as the local dynamics of available constructive cells nearby, the remaining three-dimensional tissue structures, and the degree and chronicity of prior infection [15,16,17,18,19]. The periapical milieu is adequately resourced with vasculature carrying nutrients and oxygen and a variety of cells mainly of periodontal origin, which can play a key role in regenerating tissues in the previously infected or necrotic root canal space. After the waning of tissue-destructive inflammatory processes, the root canal microstructure may function as a satisfactory framework that enhances cell adhesion and perhaps directs other cellular behaviors, if it is properly conditioned.

**Figure 1:**
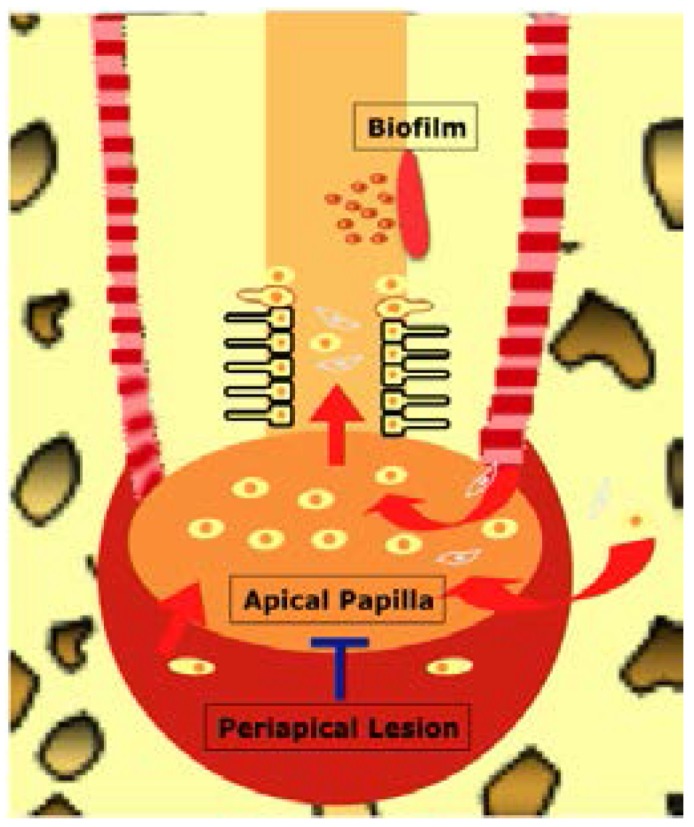
The regeneration of the pulp-dentin complex. Stem/progenitor cells at the apical tissues including stem cells of the apical papilla (SCAP), inflamed periapical progenitor cells, periodontal ligagment stem cells, and bone marrow mesenchymal stem cells should be mobilized into the root canals. Long-term infection may cause the detrimental effects on the migration and differentiation of the stem/progenitor cells. The mature resident cells of periodontal origin competitively participate in tissue healing processes and may form tissues of periodontal origin in the root canal space.

It is important to stress that the degree and chronicity of prior infection significantly affects the process of wound healing, by devastating essential host structures and available constructive cells—stem/progenitor cells and resident cells from periapical tissues and apical papilla (in immature permanent teeth) [15,16,17,18,19,20]. In order for ideal tissue healing to occur, stem cells from the periapical tissues should be mobilized into the root canals and participate in tissue regeneration (Figure 1). Long-term infection with large periapical lesions may eliminate the stem cell population near the periapical areas such as stem cells of the apical papilla (SCAP) [21]. The mesenchymal stem/progenitor cells from remote sites and inflamed periapical tissues [22] and hematopoietic stem cells might not be key players in dental pulp regeneration due to their smaller quantities and slower proliferation/differentiation potentials compared with readily available mature resident tissue-forming cells including osteoblasts, cementoblasts, and fibroblasts from periodontal ligament and alveolar bone. Therefore, it is crucial to prevent the migration of these mature resident cells of periodontal origin and stimulate the recruitment of stem/progenitor cells at the apical tissues for dental pulp-dentin regeneration [23]. Furthermore, the root canal dentin microstructures altered by chronic infection might become unfavorable for cell attachment and differentiation. The prominent microstructure that can be altered in dentin is dentinal tubules, which may be invaded by microorganisms or blocked by mineralized tissue (reparative dentin) during chronic infection [24,25]. The traumatic injury in uninfected teeth during root maturation also can alter the dentin microstructure by forming less tubular reactionary dentin therapy [26] and make the regeneration of the pulp-dentin complex more challenging during the regenerative endodontic therapy.

Several lines of evidence inferred from the findings of human and animal studies suggest that the presence of prior infection may affect the characteristics of tissues formed in the root canal space (Table 1). The histological observations from previously infected human teeth in case reports [27,28] have consistently demonstrated that tissues formed into the root canals are composed of periodontal ligament-like fibrous connective tissue and mineralized tissue integrated with the root canal dentin or scattered within the root canal space, which resembles bone and cementum. By contrast, the regeneration of the pulp-dentin complex with distinctive odontoblast-like cells along the root canal dentin was reported in an immature human tooth with irreversible pulpitis [29]. Animal studies using an infection model, which include the induction of periapical lesions, have corroborated the anecdotal evidence from human case reports [12,13,14]. Similar ectopic tissues—cementum, bone, and periodontal ligament in theses animal studies were found in the root canal space following regenerative endodontic treatment of immature canine teeth with apical periodontitis [12,13,14]. On the other hand, other animal studies revealed that pulp-like and dentin-like tissues were formed in non-infected root canals, although these studies employed more advanced tissue engineering strategies involving transplantation of highly angiogenic and neurogenic stem cell subpopulation [30,31,32,33].

## 3. Microenvironment for the Regeneration of the Pulp-Dentin Complex

A careful observation of how cells interact with biological surfaces may reveal the importance of the dentin microstructures for dental pulp-dentin regeneration. It has been well studied that the interaction between cells and the surfaces can determine cell migration [34,35,36], alignment [37,38,39,40] and cell fate [41,42]. Therefore, the microenvironment in the root canal space where cells migrate towards and interact with is critical in the regeneration of the pulp-dentin complex. Although, as discussed above, the microstructure of dentin is altered by long-term root canal infection, it may be conditioned to allow for cell attachment by chemical means. The conditioned dentin also can release endogenous growth factors, which may control cellular functions of surrounding cells [43]. However, the guidance of stem cell fate and proper cell alignment on the root canal dentin surface may not be easily attainable.

During the tooth development, odontoblasts are differentiated from the outer cells of dental papilla by signals from enamel knots and make dentin microstructures [44,45,46]. Dentinal tubules form as dentin matrix is mineralized by secretion of hydroxyapatite from odontoblastic processes [44,45,46]. These biological pores with diameters ranging from 0.9 μm to 3.0 μm result from odontoblast differentiation and dentinogenesis [47]. Therefore, one can suggest that a similar dentinogenic process could be recapitulated—in other words, stem cells differentiate into odontoblasts without the existing tubular dentin, align themselves and form dentinal tubules as well as dentin in an orderly manner—during the dentin-pulp regeneration after regenerative endodontic procedures. However, robust regeneration of tubular dentin and odontoblast alignment in regenerated dentin has rarely been shown in human and animal studies after the pulp regeneration procedure [12,13,14,27,28]. As well documented in the field of mechanical tissue engineering, the geometry of the biological surfaces can be a determinant of the alignment and fate of cells as well as cell migration [34,35,36,37,38,39,40,41,42]. Cells can sense the surface topography or geometry and adjust their alignment and differential potentials. Therefore, it can be hypothesized that dentinal tubules as a geometrical microstructure impart structural cues required for the attachment, alignment and differentiation of recruited stem cells. Although further research is needed to test this hypothesis, geometrical control of dentin microstructures by preserving or restoring tubular dentin can be a significant factor in determining the regeneration of the pulp-dentin complex and histological outcome of regenerative endodontic therapy.

**Figure 2:**
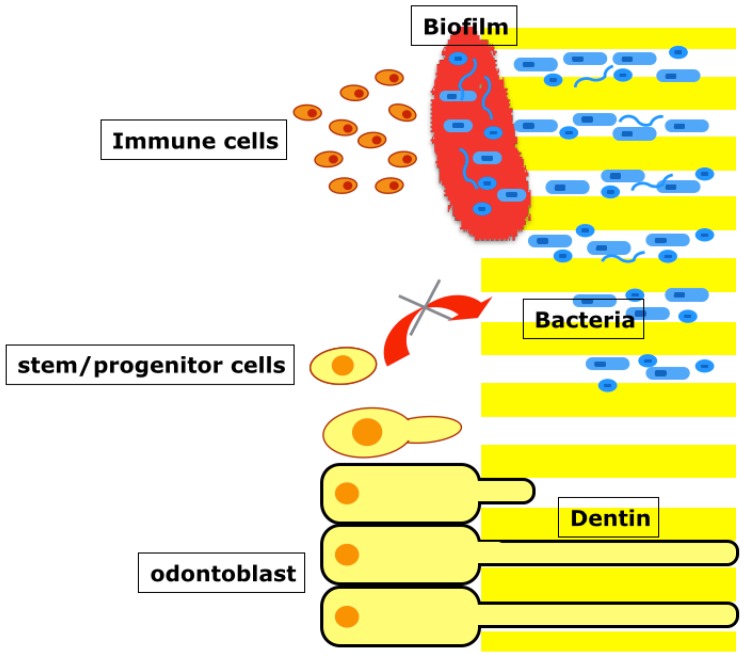
The presence of of bacteria mounts host immune defenses and diverts the tissue healing process towards repair rather than regeneration. The infected dentin surface does not allow for cell adhesion and cannot provide an adequate geometrical cue to the mobilized cells in main canals.

In chronic infection, microogranisms can invade the dentinal tubules and survive the chemomechanical preparation of root canals [24,25]. Moreover, biofilms integrated into dentin are considered the most recalcitrant type of chronic root canal infection and often exist in the root canal intricacies even after mechanical debridement [48,49,50]. This infected dentin surface does not allow for cell adhesion and cannot provide an adequate geometrical cue to the mobilized cells in main canals. Even if cells manage to migrate to the dentin surface, the host mobilizes the immune defense system and mounts an inflammatory response to the bacteria (Figure 2). It is presumed that these inflammatory processes can significantly interfere with the proliferation and differentiation processes of stem cells and may allow the resident cells to rapidly repair the injury site with soft and hard tissues of periodontal origin—periodontal ligament, cementum, and bone as bacteria are removed and inflammation subsides.

## 4. Clinical Disinfection Protocols for Dental Pulp Regeneration

In order for regeneration to occur in an uninterrupted and coordinated manner, the root canal microenvironment must be properly prepared and disinfected. American Association of Endodontists (AAE) has published “Clinical Considerations for a Regenerative Procedure” [51] based on the best current available evidence, although most of the available evidence regarding the disinfection regimen is based on *in vitro* findings. Key points from this guideline with regards to disinfection include use of lower concentration of sodium hypochlorite (1.5%) followed by saline or ethylene diamine tetraacetic acid (EDTA) as a chemical disinfectant [52] and calcium hydroxide or low concentration of triple antibiotic paste (0.1 mg/mL) as a intracanal medicament [53] at the first appointment, and use of EDTA [54] as a single irrigant at the second appointment.

A recent study by Kontakiotis *et al.* [55] based on 60 clinical trials has revealed a striking heterogeneity in their disinfection protocols among studies, although most of the included studies in the review have showed clinically successful outcomes. Clinical outcome studies using different clinical disinfection protocols have reported success rates ranging from 85% to 100% [7,8,9,10,11]. Many clinical studies, unlike the AAE guideline, employed high concentrations of sodium hypochlorite (2.5%–6%) and high concentrations of antibiotic pastes at the first appointment and sodium hypochlorite instead of EDTA at the second appointment [55]. Interestingly, approximately 32% of studies used mechanical instrumentation, and 24% of studies used chlorhexidine at the first appointment. Of note, chlorhexidine and mechanical instrumentation are not suggested in the AAE guideline [51]. Lin *et al.* [56] reported a failed case due to the presence of biofilms in the apical root canal surviving chemical disinfection even when chlorhexidine, high concentration of sodium hypochlorite (5.25%), calcium hydroxide, and triple antibiotic paste were used, suggesting the necessity of mechanical debridement.

On the basis of consistently high success rates in clinical trials using dissimilar protocols [7,8,9,10,11], one can argue that disinfection protocols are not critical for clinical success and our conventional chemomechanical preparation is sufficient to provide pulp regeneration. However, the clinical outcome cannot be interpreted as the histological success, *i.e.*, regeneration of pulp-dentin complex, which may require more advanced disinfection protocols.

## 5. Advanced Disinfection Techniques

**Figure 3:**
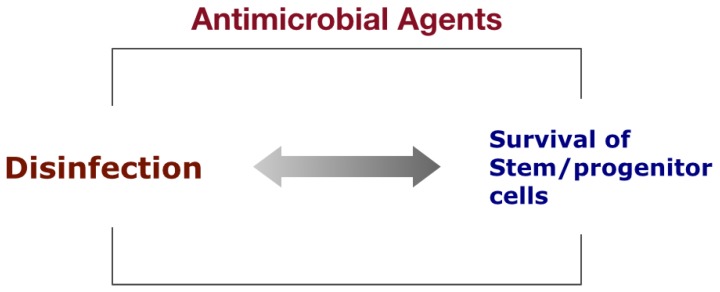
Sufficient disinfection and survival of stem/progenitor cells at the apical tissues cannot be reliably achieved with non-selective conventional disinfectants.

Conventional disinfectants such as sodium hypochlorite and antibiotic pastes cannot reliably achieve two primary goals considered vital to successful dentin and pulp regeneration at the same time—sufficient disinfection and survival of stem/progenitor cells at the apical tissues (Figure 3). At least 5-log bacterial reduction (99.999%) seems adequate for the root canal microenvironment conducive to regeneration, but most of the conventional disinfection protocols are ineffective in reducing the number to this level (less than 3-log reduction) [57,58,59,60]. Furthermore, conventional agents do not selectively target the pathogens in the root canal but also kill host stem/progenitor cells at the apical tissues to be mobilized into the root canal space.

Recently, new disinfection methods have been developed to overcome the limitations of conventional disinfecting protocols. These advanced techniques such as photon-induced photoacoustic streaming (PIPS) [60,61], nanoparticles [62], photodynamic therapy [63], and multisonic disinfection system [64] as well as cavitation-generating devices [65] not only reduce planktonic bacteria in the root canal system but also target biofilms by either disrupting the biofilm structure or killing bacteria within the biofilms. The cavitation-generating devices, PIPS, and multisonic disinfection system can disrupt biofilms and make bacteria more vulnerable to disinfectants, while nanoparticles and photodynamic therapy allow the molecules to penetrate into the biofilm structure and kill bacteria. Although these newer disinfection strategies may allow for enhanced antimicrobial efficacy within the root canal system, their toxicity to stem/progenitor cells at the apical tissues has not been investigated yet.

### 5.1. Cavitation-Generating Devices (Sonic or Ultrasonic Devices)

**Figure 4:**
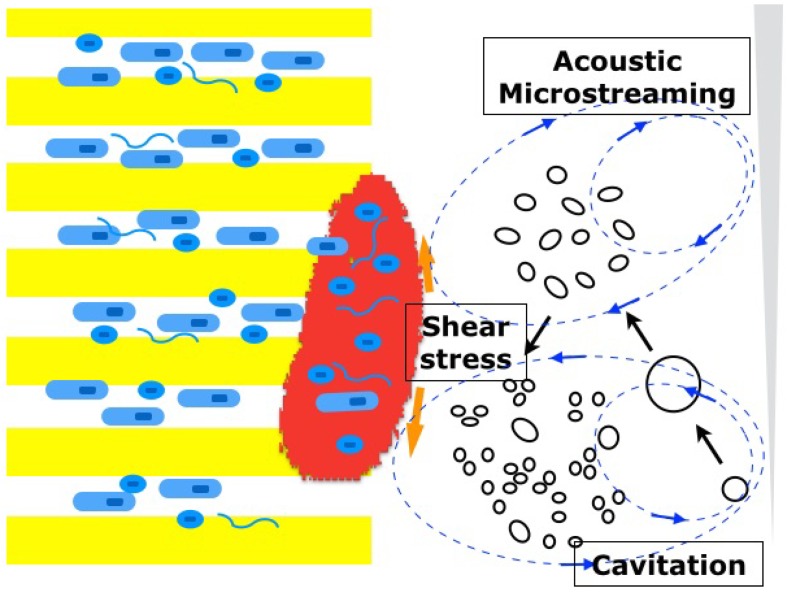
Cavitation-generating devices. Inertial cavitation bubbles are generated by sonic or ultrasonic devices and expand and collapse into smaller bubbles. During the bubble implosion, a large amount of shear stress is produced (**cavitation, black circles**). Rapid circular movements of liquid around the sonic or ultrasonic tips can also create shear stress (**acoustic microstreaming, blue lines**).

Sonic devices can generate inertial cavitation bubbles in a liquid medium, which slowly expand and rapidly collapse into smaller bubbles [66]. During this bubble implosion, a large amount of energy is created to cause shear stress to the surrounding structure (root canal dentin) [66] (Figure 4). The process is called cavitation, and its force is proportional to the number of the inertial bubbles [66]. On the other hand, ultrasonic devices generate mostly non-cavitation bubbles and induce acoustic microstreaming [67] that allows the directional circular movements of fluid within the root canals, but the shear stress created by this mechanism is much smaller compared with that of cavitation. Interestingly, the introduction of microbubble emulsion in the medium was found to be effective against biofilms at the apical root canals by means of the increased cavitation force even when ultrasonic devices were used [65].

### 5.2. Photon-Induced Photoacoustic Streaming (PIPS)

**Figure 5:**
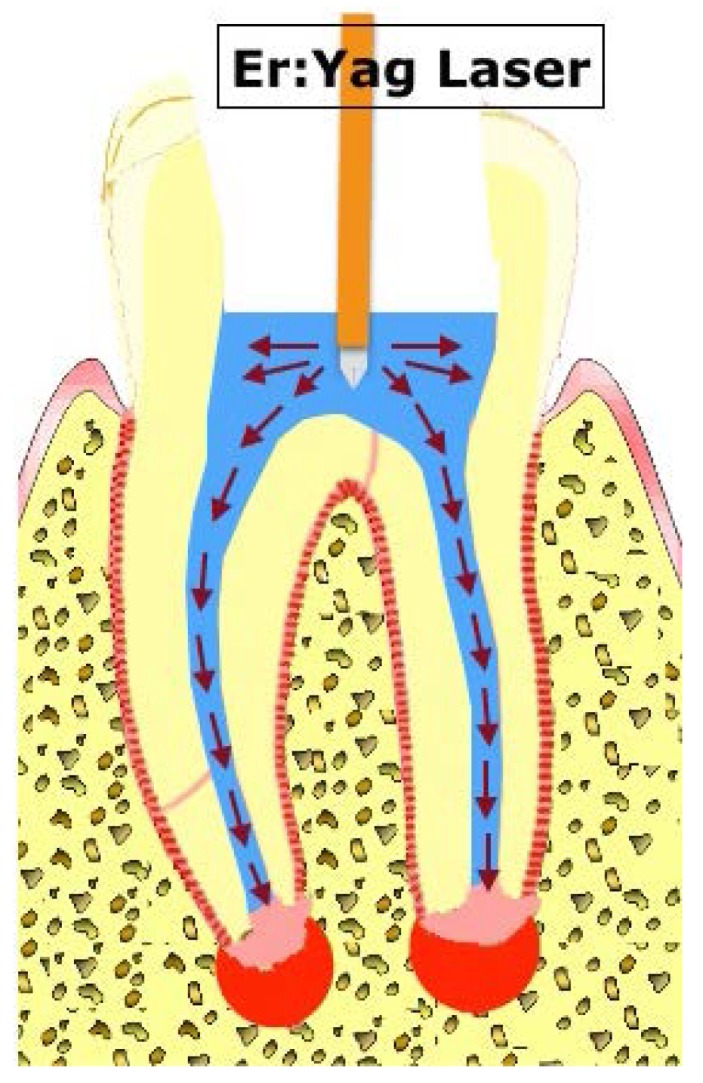
Photon-induced photoacoustic streaming. Low-power Er:Yag lasers generate vaporized bubbles around the laser tip. The bubbles implode during the laser cooling cycle and release shear stress. Cyclical shock waves (**red arrows**) are produced and can disrupt biofilms.

Low-power Er:Yag lasers are known to generate vaporized bubbles around the laser tip when their energy is absorbed by a liquid medium, in which the bubbles implode during the laser cooling cycle [60]. The implosion of the vaporized bubbles releases shear stress in a manner similar to cavitation, which can disrupt the biofilm structure. This cyclic activation of low-power (sub-ablative) lasers creates cyclical shock waves [60], which also may enhance the biofilm disruption (Figure 5). PIPS was shown to be more effective than ultrasonic devices in reducing bacteria and biofilms at the apical root canals [61].

### 5.3. Multisonic Disinfection System

**Figure 6:**
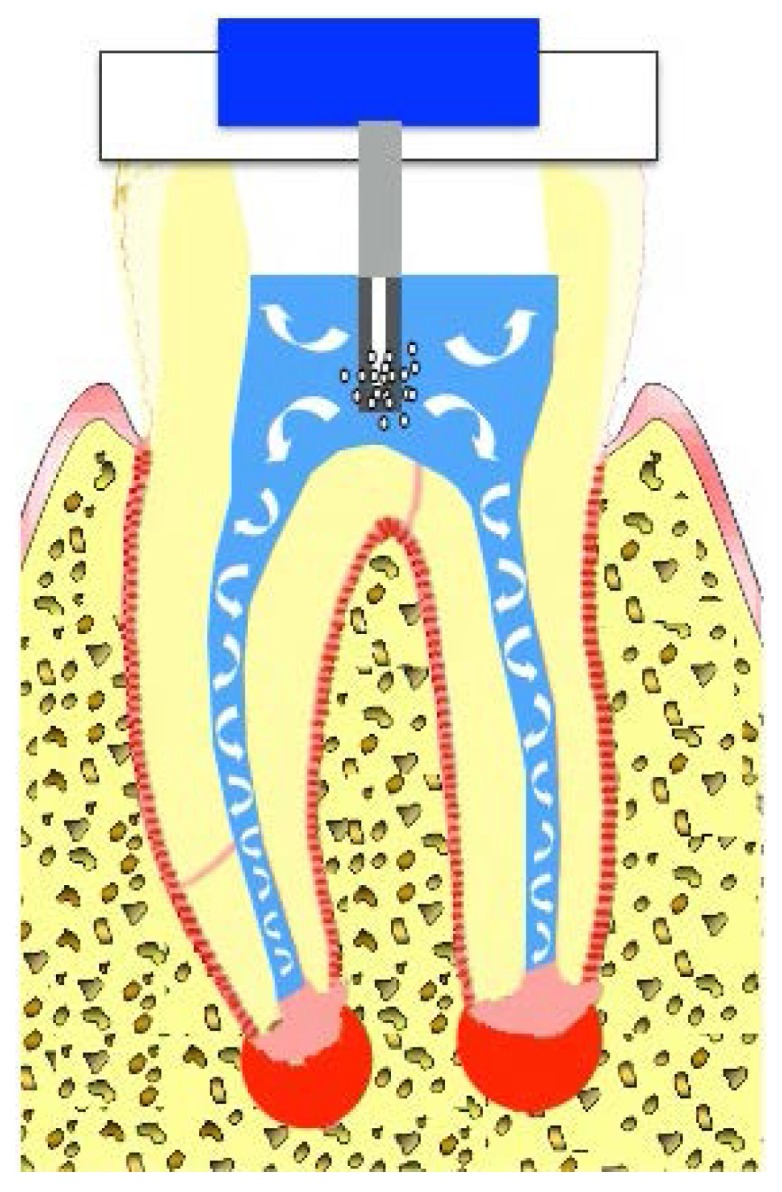
Multisonic disinfection system. A cloud of inertial cavitation microbubbles release a significantly higher cavitation force compared with those of sonic devices during the bubble implosion.

The multisonic disinfection system utilizes a broad range of sonic frequencies and creates a cloud of inertial cavitation microbubbles [64]. It can generate a significantly higher cavitation force than sonic devices because a considerably larger number of inertial bubbles are created by this system (Figure 6). A study comparing the effectiveness of different irrigating devices in tissue dissolution showed that this multisonic disinfection system had significantly faster tissue dissolution compared with ultrasonic agitation, apical negative pressure irrigation, and conventional needle irrigation under the same conditions of temperature (21 °C and 40 °C) and irrigants (sterile water or 0.5%, 3%, 6% sodium hypochlorite)[60]. Notably, this system showed more rapid tissue dissolution regardless of concentrations of sodium hypochlorite compared with other irrigation methods [64].

### 5.4. Nanoparticles

**Figure 7:**
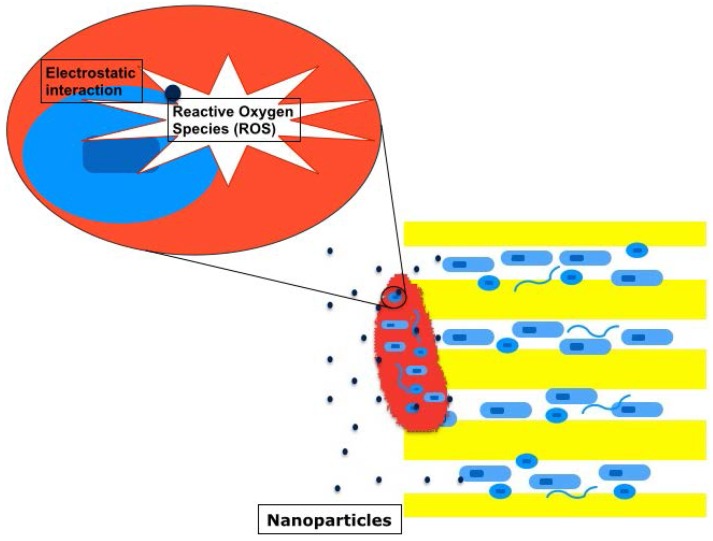
Nanoparticles. Nanoparticles (blue dots) bind to bacterial cell walls and create reactive oxygen species such as superoxide (O_2_^−^) and hydroxyl radicals (OH^−^) or induce electrostatic interaction. Nanoparticles can infiltrate the biofilm structure and kill bacteria within it.

Nanoparticles with diameters ranging from 1–100 nm can easily penetrate into dentinal tubules (diameter, 0.9 μm–3.0 μm). These ultrafine particles bind to bacterial cell walls and create reactive oxygen species such as superoxide and hydroxyl radicals or induce electrostatic interaction [62]. Negatively charged nanoparticles (silver and zinc oxide) can cause oxidative stress to bacteria by creating this reactive oxygen species [68,69], while positively charged nanoparticles (chitosan) can disrupt negatively charged bacterial cell walls by electrostatic interaction [70] (Figure 7). An observation by using confocal scanning microscopy showed that both zinc oxide and chitosan nanoparticles could infiltrate the biofilm structure (seven-day-old *E. faecalis* biofilm) and significantly reduced bacteria within it [71].

### 5.5. Photodynamic Therapy

**Figure 8:**
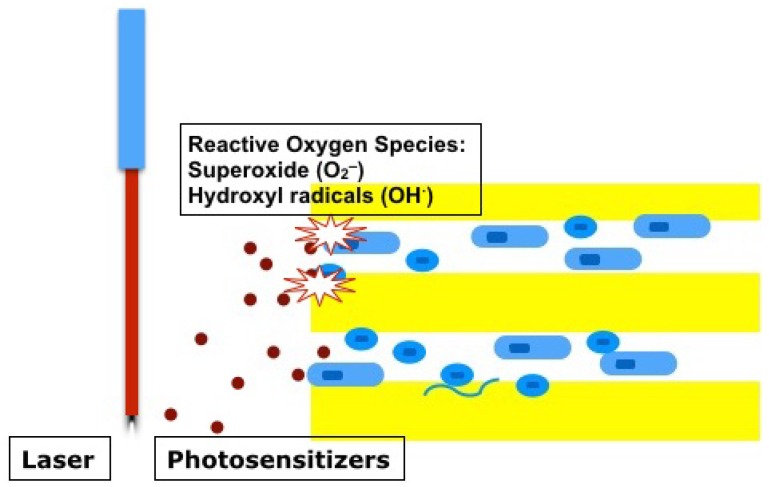
Photodynamic therapy. Lasers or LED can activate photosensitizers (red dots) and create reactive oxygen species (O_2_^−^, OH^−^). This oxidative stress kills bacteria in the dentinal tubules.

A specific wavelength light irradiation using diode lasers (660 nm or 665 nm) or light emitting diodes (LED) can activate photosensitizers such as methylene blue, toluidine blue, and Rose Bengal, and create reactive oxygen species, which cause oxidative stress to the surrounding cells in the same manner as negatively charged nanoparticles [63,72,73] (Figure 8). The conjugation of positively charged chitosan with negatively charged photosensitizers for photodynamic therapy could enhance negatively charged bacterial cell wall attachment. Furthermore, electrostatic interaction between chitosan and bacterial cells could induce the disruption of bacterial cell walls and cell death. Indeed, chitosan-conjugated Rose Bengal has shown significantly higher antibacterial effectiveness against bacterial biofilm structures (seven-day-old *E. faecalis* and *P. aeruginosa*) compared with negatively charged Rose Bengal or positively charged methylene blue [73].

## 6. Conclusions

Current conventional disinfection protocols have provided successful clinical outcomes. However, they have not allowed for the regeneration of the pulp-dentin complex in teeth with prior infection. This may be due to the limitations of conventional antimicrobial regimens. The non-selective conventional disinfectants may neither reduce the number of bacteria to a satisfactory level nor minimize the toxicity to periapical stem/progenitors. Furthermore, the microenvironment of root canals following root canal disinfection can negatively impact the fate and functions of recruited stem/progenitor cells. Dentinal tubules may provide a structural cue to the mobilized stem/progenitor cells from the apical tissues and dictate their alignment and differentiation. The presence of bacteria within dentinal tubules and biofilms surviving conventional chemomechanical disinfection poses a significant threat to the fate of the cells and may divert the tissue healing process towards repair rather than regeneration, resulting in the formation of tissues of periodontal origin. Several advanced disinfection tools such as cavitation-generating devices, PIPS, multisonic disinfection system, nanoparticles, and photodynamic therapy have been developed to overcome the limitations of the conventional disinfectants. They appear to have greater antimicrobial efficacy against chronic infection; however, further studies are warranted to investigate their effect on periapical stem/progenitor cells.

## Figures and Tables

**Table 1:** Histological outcomes after regenerative endodontic treatment in animal and human studies.

Study	Type of Study	Prior Infection	Pulp-dentin Complex	Ectopic Tissues
Wang *et al.* [12]	Animal Study	Yes	No	Yes
Gomes-Filho *et al.* [13]	Animal Study	Yes	No	Yes
Becerra *et al.* [27]	Case Report	Yes	No	Yes
Lei *et al.* [28]	Case Report	Yes	No	Yes
Saoud *et al.* [14]	Animal Study	Yes	No	Yes
Iohara *et al.* [30]	Animal Study	No	Yes	No
Ishizaka *et al.* [31]	Animal Study	No	Yes	No
Shimizu *et al.* [29]	Case Report	No	Yes	No
Iohara *et al.* [32]	Animal Study	No	Yes	No
Iohara *et al.* [33]	Animal Study	No	Yes	No
